# Successful Surgery for Type III Colonic Atresia Using the Side-to-Side Santulli Procedure: Rescue Treatment During the Practice of Global Pediatric Surgery

**DOI:** 10.7759/cureus.102914

**Published:** 2026-02-03

**Authors:** Haruka Kobayashi, Shin Miyata, Johann Paulo Guzman, Satoshi Ieiri, Domingo T Alvear

**Affiliations:** 1 Digestive and General Surgery, 35th Medical Group - Misawa AFB Medical Facility, Misawa, JPN; 2 Pediatric Surgery, SSM Health Cardinal Glennon Children's Hospital, St. Louis, USA; 3 College of Medicine, Visayas State University, Baybay, PHL; 4 Pediatric Surgery, Kagoshima University, Kagoshima, JPN; 5 Surgery, World Surgical Foundation, City of Manila, PHL

**Keywords:** ascending colostomy, caliber difference, colonic atresia, global surgery, santulli procedure, stoma prolapse

## Abstract

Colonic atresia (CA) is a rare congenital intestinal condition that requires surgical intervention. The surgical approaches for its treatment may vary from primary anastomosis to stoma creation. We report a case of CA treated with side-to-side anastomosis and Santulli-type colostomy. A three-year-old girl with a history of CA who had undergone ascending colostomy in the neonatal period visited the surgical mission of the World Surgical Foundation for definitive surgical management. The details of the operative findings in the neonatal period are unknown. Preoperative contrast enema revealed an unused rectosigmoid segment, suggesting that primary end-to-end anastomosis would be challenging. Exploratory laparotomy revealed type III CA in the ascending colon. The distal colon, from the transverse to the descending colon, was absent. Due to the 5-fold caliber difference between the ascending and sigmoid colon, a side-to-side Santulli procedure with an end colostomy was performed to restore colonic continuity. The procedure effectively restored intestinal continuity and addressed a significant caliber discrepancy. Postoperative recovery was uneventful, with consistent colostomy output and normal rectal stool defecation. Two months postoperatively, the patient remained in a good general condition and had a favorable bowel function.

In conclusion, the side-to-side Santulli procedure offers a safe approach for CA, providing an additional safeguard as a pressure-release valve for cases with significant caliber differences between the proximal and distal colon, ensuring smooth stool passage.

## Introduction

Colonic atresia (CA) is a rare congenital anomaly of the gastrointestinal tract with an incidence ranging from 1 in 10,000 to 66,000 live births [1]. The etiology of this anomaly is thought to be of vascular origin, and there is a slight male predominance in prevalence [2]. The most common type is type III (90%), with 38% of cases occurring in the right colon [3]. It typically presents 24 to 48 hours after birth with gradual abdominal distension, inability to pass meconium, and bilious or feculent emesis [3]. Prompt surgical intervention is imperative to address CA; however, lack of access to care can become a major source of morbidity and mortality in neonates with CA. In the Philippines, particularly on smaller islands, a shortage of surgical treatment by pediatric surgeons means that many children wait for surgery.

The World Surgical Foundation (WSF) provides assistance once a year. During a surgical mission with the WSF, we encountered the case of a three-year-old girl with CA who initially underwent a colostomy created by a general surgeon and suffered from a subsequent stoma prolapse [4]. We herein report a case of successful rescue surgery for CA treated with bowel-preserving side-to-side anastomosis and Santulli-type colostomy.

## Case presentation

We encountered a three-year-old girl with a history of CA who underwent ascending colostomy in the neonatal period.

Neonatal history

The patient presented with delayed passage of meconium and bilious vomiting during the neonatal period. Radiographic assessment revealed severe dilation of both the small and large bowels, suggesting a distal bowel obstruction. On the third day of life, exploratory laparotomy was performed by a local general surgeon. Intraoperatively, type III CA in the ascending colon was identified, and an ascending colostomy was created. The patient had no associated cardiac, urogenital, or skeletal anomalies, and a patent anal opening was confirmed. Further details of the operative findings in the neonatal period were unavailable. She subsequently developed stoma prolapse.

Current history

**Figure 1:**
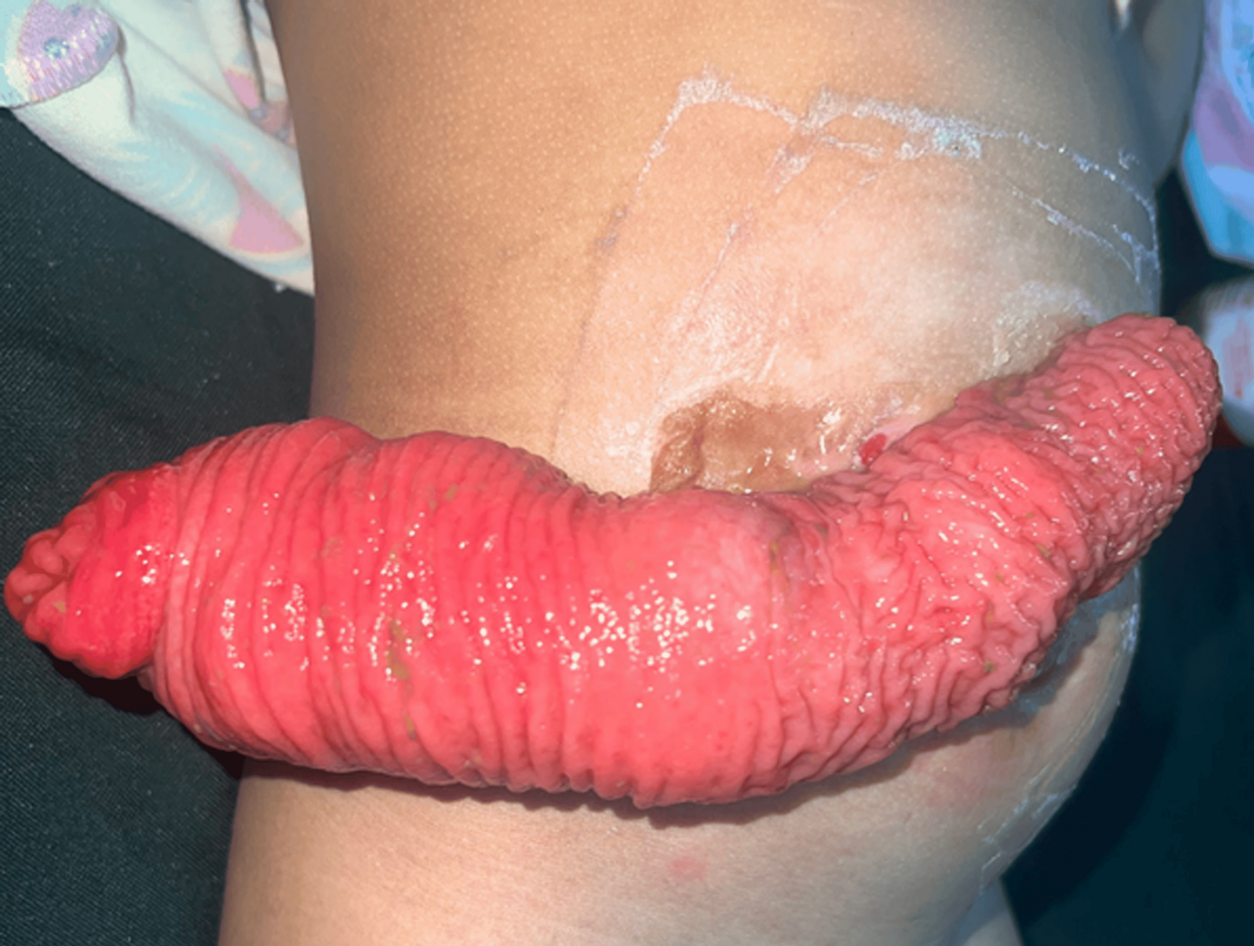
Appearance of prolapsed ascending colostomy

**Figure 2:**
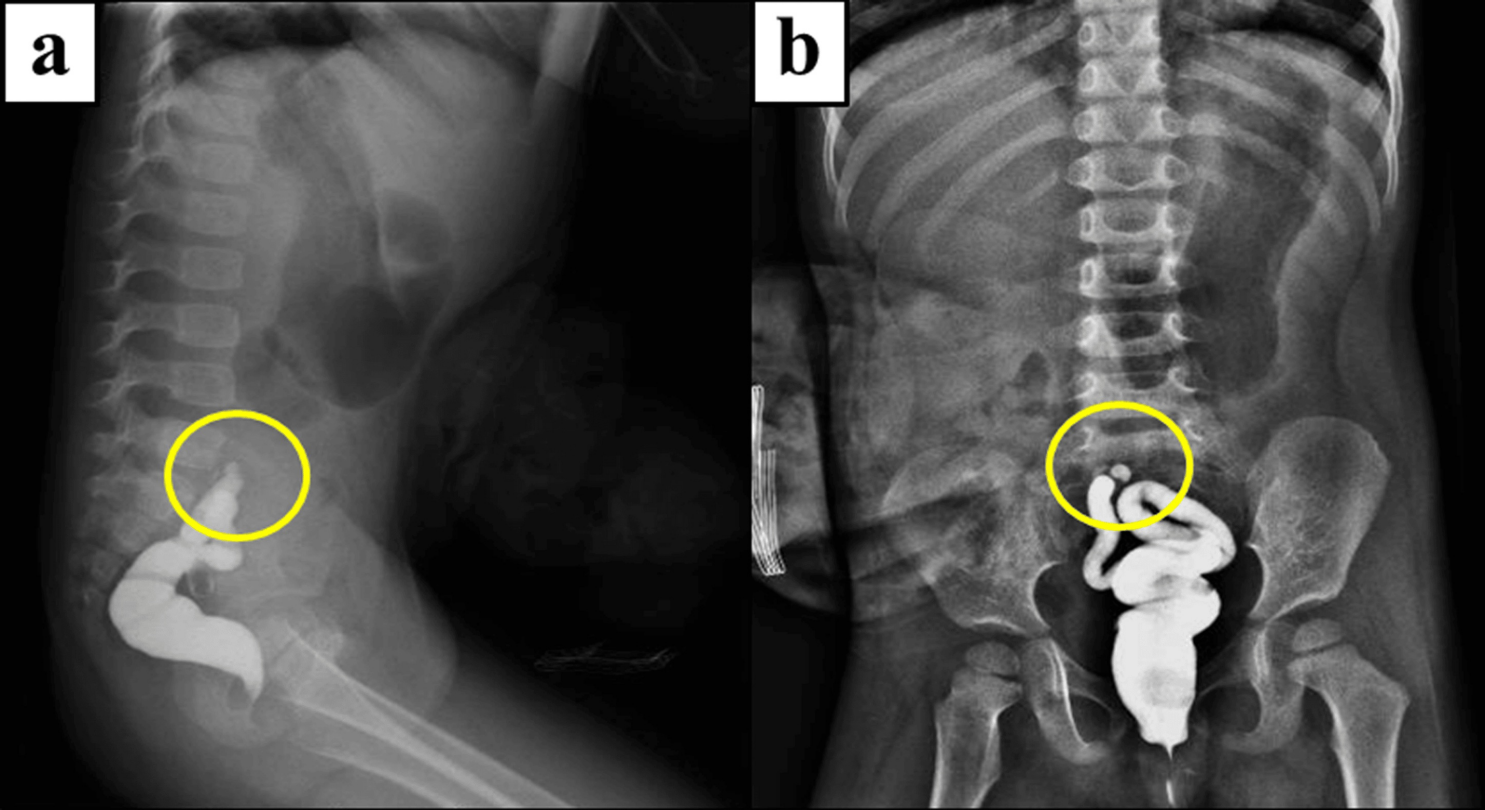
Preoperative contrast enema a: Lateral view; b: AP view

During a surgical mission of the WSF in the Philippines, the patient presented to our screening for definitive surgical management. She had a history of stoma prolapse (Figure 1). A preoperative contrast enema revealed a short microcolon, as shown in Figure 2.

Operative procedure

**Figure 3:**
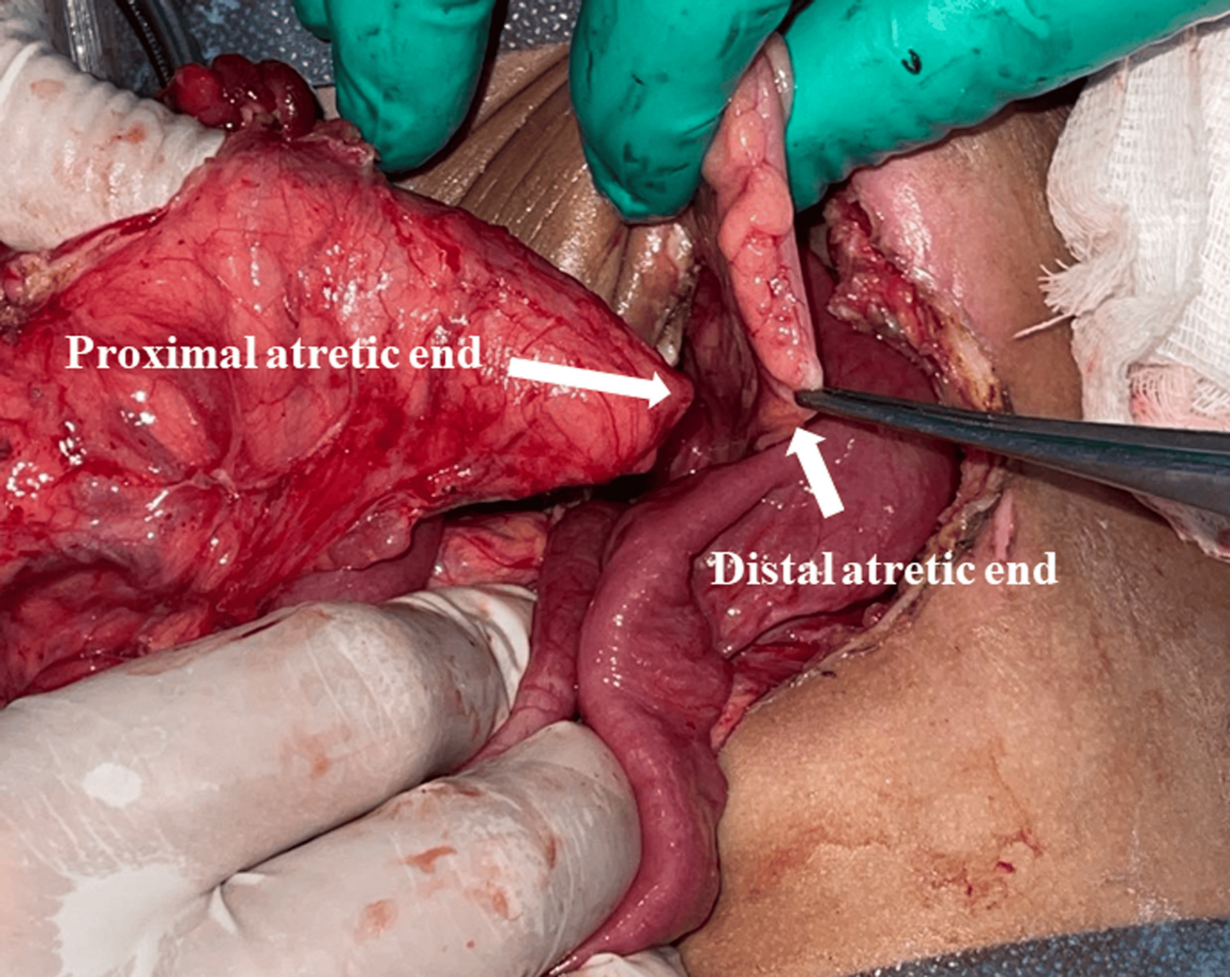
Operative findings A size mismatch of the bowel caliber was recognized between the ascending colon and the atrophic sigmoid colon

**Figure 4:**
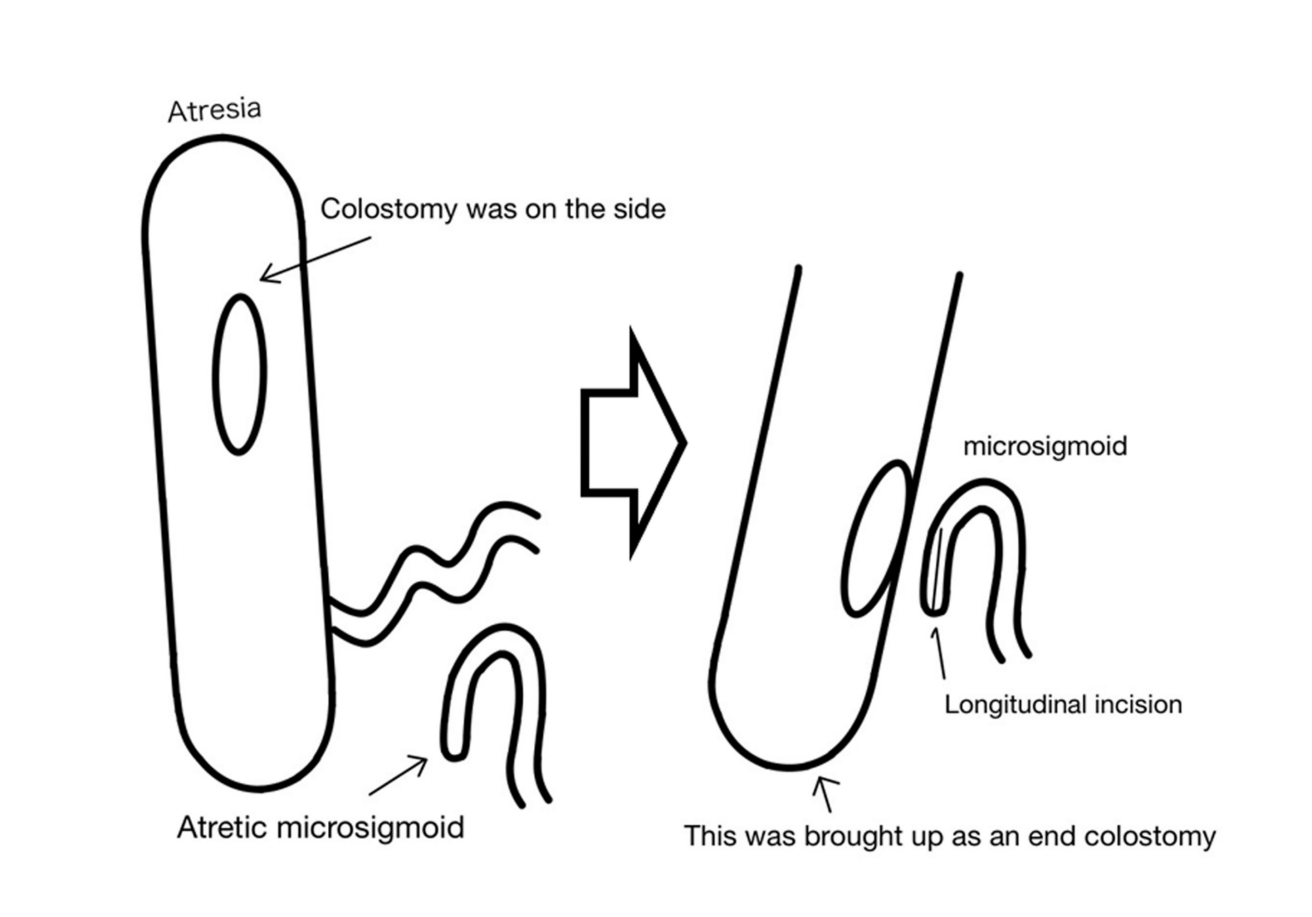
Schematic illustration of the operative findings

**Figure 5:**
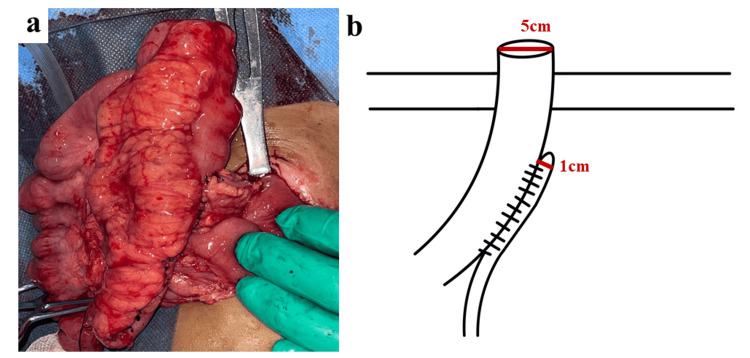
The procedure performed in the present case a: Operative findings of the side-to-side Santulli procedure; b: Schematic illustration of the side-to-side Santulli procedure

Under general anesthesia, the prolapsed ascending colostomy was successfully reduced manually. Upon entering the abdominal cavity and after mobilization of the colostomy, a blind-ending ascending colon and another blind-ending microrectosigmoid colon were identified. The transverse colon and descending colon were not present (Figure 3), possibly because of either resection of these segments during the initial surgery or a rare congenital defect in which the segment between the blind ends was absent, resulting in a long gap. Owing to the absence of records from the initial surgery, determining the exact cause was not feasible in this case. A substantial bowel caliber discrepancy between the ascending colon and sigmoid colon was noted, estimated to be approximately 1:5, as shown in Figures 3, 4. To resolve this problem while achieving bowel continuity, we decided to employ the side-to-side Santulli procedure with an end colostomy using the proximal ascending colon and unused sigmoid colon, as shown in Figures 4, 5.

Postoperative course

After surgery, the patient exhibited a favorable clinical course characterized by a normal bowel function with stool output from both the colostomy and the anus, and there were no signs of leakage or other postoperative complications. A two-month follow-up examination revealed smooth recovery. Colostomy closure will be planned after sufficient conditioning of the unused colon, which will be confirmed by a contrast enema.

## Discussion

We report a successful rescue surgery using the Santulli procedure in a case of CA characterized by a significant size discrepancy between the distal and proximal bowel, approximately 1:5.

Colorectal atresia requires surgery to establish intestinal continuity. Such procedures should avoid complications, such as anastomotic leakage, stenosis, or obstruction. Obstruction can be either mechanical or functional. Surgical techniques designed to restore bowel continuity while preserving an ostomy as a safeguard against inadequate stool passage through the site of anastomosis have been collectively termed the ostomy in continuity approach; the Santulli procedure is one such technique [5].

The Santulli procedure traditionally involves creating an end-to-side anastomosis between the proximal and distal bowel segments, with the proximal bowel also removed as an ostomy. This setup allows the stool to pass into the distal bowel, while the ostomy acts as a pressure release, safeguarding against potential complications from increased intraluminal pressure or anastomotic dysfunction. The Santulli procedure also restores enterohepatic circulation, preserves enterohepatic flora, mitigates the risk of bypass colitis, and reduces the likelihood of biliary stasis, sodium deficiency, and metabolic acidosis, particularly in cases of short bowel syndrome [6]. Furthermore, it helps maintain the gut microbiota, prevents intestinal stacking, and facilitates the rapid restoration of the motility and function of the bowel following stoma closure. The Santulli procedure aligns with the concept of gradually stimulating the dysfunctional distal bowel and facilitating the perfusion of nutrients and stool to improve gastrointestinal autonomy [6]. For cases with substantial caliber discrepancies, a side-to-side modification of Santulli anastomosis can further optimize stool passage while retaining the ostomy pressure-release function [3].

In the present case, the proximal colon measured approximately 7.5 cm in diameter and the distal colon 1.5 cm, corresponding to a ratio of 5:1, which is generally considered a significant challenge for primary anastomosis. Although primary anastomosis remains the standard when the discrepancy is acceptable, no strict cutoff exists, and a 3:1 ratio is often cited in the literature as a threshold [7,8]. To clarify our decision-making process, we summarized the advantages, limitations, and applicability of different surgical options for intestinal atresia with caliber discrepancy (Table 1) [6-10]. Based on this comparison, we considered the Santulli procedure the safest option in this setting, as it balanced decompression, distal stimulation, and feasibility in a resource-limited environment.

**Table 1:** Surgical options for intestinal atresia with a significant caliber discrepancy This table summarizes the major procedures for managing intestinal atresia with a significant proximal-to-distal caliber discrepancy.

Procedure	Advantages	Limitations	Resource-Limited Settings
Primary anastomosis [7,8]	Single stage; physiologic	Risk of obstruction with severe caliber gap; no safety outlet. Although no standard cutoff, an ~3:1 ratio is often cited	Standard when discrepancy is acceptable
Temporary diverting stoma (DS) [9]	Simple; decompresses; stabilizes	Distal bowel unused until closure; long total parenteral nutrition (TPN); requires second surgery	Highly feasible; widely used
Santulli procedure [6,10]	Decompression; distal continuity; caliber adaptation	Requires second surgery; stoma output may be high	Favorable; useful in low-resource sites
Bishop–Koop enterostomy [9]	Decompression; caliber adaptation, “test-drive” distal bowel; small stoma	Requires second surgery; stoma output may persist	Favorable; useful in low-resource sites

Unlike primary anastomosis for treating CA, Santulli enterostomy requires a secondary procedure for colostomy closure; however, this closure is generally straightforward and requires minimal bowel resection. Delayed interventions often contribute to significant morbidity and mortality in areas with limited access to pediatric surgical care. For temporary surgical missions in regions where pediatric surgeons and specialized resources may be scarce, the Santulli procedure offers a practical alternative for congenital pediatric bowel anomalies. Colostomy closure is technically uncomplicated, allowing even general surgeons to perform it effectively when specialized care is unavailable.

## Conclusions

The side-to-side Santulli procedure represents a safe and effective bowel-preserving strategy for pediatric patients with CA, particularly in the presence of a marked caliber discrepancy between the proximal and distal bowel segments. By combining restoration of intestinal continuity with a functional pressure-release mechanism, this approach minimizes the risks of anastomotic leakage, obstruction, and functional failure. In the present case, the side-to-side modification of the Santulli anastomosis allowed smooth stool passage despite a severe 5:1 caliber discrepancy, while simultaneously promoting gradual functional adaptation of the unused distal colon.

In addition to its physiological advantages, the Santulli procedure offers practical benefits in resource-limited settings, where delayed presentation and limited access to specialized pediatric surgical care are common. The technique provides a staged and safer pathway to definitive reconstruction, with a subsequent stoma closure that is technically straightforward and does not require extensive bowel resection. Therefore, the side-to-side Santulli procedure should be considered a valuable and versatile surgical option for complex cases of CA, especially during humanitarian surgical missions or in environments where surgical resources and follow-up capabilities are constrained.
